# Randomised controlled trial of an automated, interactive telephone intervention to improve type 2 diabetes self-management (Telephone-Linked Care Diabetes Project): study protocol

**DOI:** 10.1186/1471-2458-10-599

**Published:** 2010-10-12

**Authors:** Dominique Bird, Brian Oldenburg, Mandy Cassimatis, Anthony Russell, Susan Ash, Mary D Courtney, Paul A Scuffham, Ian Stewart, Richard Wootton, Robert H Friedman

**Affiliations:** 1 Department of Epidemiology and Preventive Medicine, School of Public Health and Preventive Medicine, Monash University, 3rd Floor Burnett Building The Alfred Hospital, Melbourne, 3004, Australia; 2 Centre for Online Health, University of Queensland, Brisbane, Australia; 3 Diamantina Institute, University of Queensland, Princess Alexandra Hospital, Brisbane, Australia; 4 Department of Diabetes and Endocrinology, Princess Alexandra Hospital, Brisbane, Australia; 5 Institute of Health and Biomedical Innovation, Queensland University of Technology, Brisbane, Australia; 6 Faculty of Health and Social Development, University of British Columbia, Vancouver, Canada; 7 Centre for Applied Health Economics, School of Medicine, Griffith University, Logan, Australia; 8 Norwegian Centre for Integrated Care and Telemedicine, University Hospital of North Norway, Tromsø, Norway; 9 Medical Information Systems Unit, Boston Medical Center, Boston University, Boston, USA

## Abstract

**Background:**

An estimated 285 million people worldwide have diabetes and its prevalence is predicted to increase to 439 million by 2030. For the year 2010, it is estimated that 3.96 million excess deaths in the age group 20-79 years are attributable to diabetes around the world. Self-management is recognised as an integral part of diabetes care. This paper describes the protocol of a randomised controlled trial of an automated interactive telephone system aiming to improve the uptake and maintenance of essential diabetes self-management behaviours.

**Methods/Design:**

A total of 340 individuals with type 2 diabetes will be randomised, either to the routine care arm, or to the intervention arm in which participants receive the Telephone-Linked Care (TLC) Diabetes program in addition to their routine care. The intervention requires the participants to telephone the TLC Diabetes phone system weekly for 6 months. They receive the study handbook and a glucose meter linked to a data uploading device. The TLC system consists of a computer with software designed to provide monitoring, tailored feedback and education on key aspects of diabetes self-management, based on answers voiced or entered during the current or previous conversations. Data collection is conducted at baseline (Time 1), 6-month follow-up (Time 2), and 12-month follow-up (Time 3). The primary outcomes are glycaemic control (HbA1c) and quality of life (Short Form-36 Health Survey version 2). Secondary outcomes include anthropometric measures, blood pressure, blood lipid profile, psychosocial measures as well as measures of diet, physical activity, blood glucose monitoring, foot care and medication taking. Information on utilisation of healthcare services including hospital admissions, medication use and costs is collected. An economic evaluation is also planned.

**Discussion:**

Outcomes will provide evidence concerning the efficacy of a telephone-linked care intervention for self-management of diabetes. Furthermore, the study will provide insight into the potential for more widespread uptake of automated telehealth interventions, globally.

**Trial Registration Number:**

ACTRN12607000594426

## Background

Diabetes is a leading cause of death and morbidity and is a health priority in Australia and worldwide. Over 285 million people have diabetes around the world [1] (90% of whom are diagnosed with type 2 diabetes). For the year 2010, it is estimated that 3.96 million excess deaths in the age group 20-79 years are attributable to diabetes around the world [2]. The number of people living with diabetes is predicted to reach 439 million in 2030 [1]. Poor glycaemic control, as measured by Haemoglobin A1c (HbA1c), significantly increases one's risk of costly diabetes-related complications [3,4].

Self-management is an integral component of effective diabetes care [5]. Systematic reviews of interventions targeting diabetes self-management indicate that the effectiveness of these programs is significantly related to their duration [6,7]. This emphasises the importance of ongoing follow-up and support for successful long-term glycaemic control [8].

The delivery of ongoing support to the fast growing number of people living with diabetes presents a real challenge for most health systems and this will not be addressed by any modest increase in the number of relevant health professionals to provide such services. Addressing this challenge requires new cost-effective approaches that will reach a large number of individuals regularly, in particular, those people who already have poor access to current services, due to geographical, financial or other barriers.

Programs using automated information and telecommunication technologies offer a potential solution to chronic disease management as they can be conveniently accessed from home or office and at any time of the day or night. Reviews of such interactive automated technologies in chronic disease care, including diabetes [9-11], report positive effects on users' self-care knowledge, clinical and behavioural outcomes, social support and health care utilisation. In addition, acceptability of automated telephone programs among users has been shown to be high [12]. Further, the logical structure, on which computerised interventions are built, means that they offer a consistency of delivery which is difficult to achieve in programs delivered by health professionals. Therefore, these technologies hold promise as a new approach to overcome barriers associated with the traditional delivery of chronic disease self-management programs by offering effectiveness, accessibility and consistency. They may also prove to be cost-effective.

The use of a telephone as the mode of access to these programs makes them available to the majority of the population. The Telephone-Linked Care (TLC) system is an interactive computer assisted telephone system which has been shown to improve health behaviours and to be acceptable to users [13-17]. This system consists of a computer connected to the telephone network, equipped with speech recognition, numerous pre-recorded conversation statements and a database in which users' answers are stored. It is designed to emulate telephone conversations between patients and health professionals. It tailors its responses, including feedback and encouragement, according to data entered in the TLC database and the answers that it receives during the current and previous calls.

This paper presents the study protocol for a randomised controlled trial (RCT) of a TLC system aiming to improve type 2 diabetes management, TLC Diabetes. We hypothesise that participants in the intervention arm will demonstrate greater improvements in HbA1c and health-related quality of life (QoL) compared to those in the control arm. Secondly, we hypothesise that the intervention will be cost-effective compared with the control arm. The results of this study will provide valuable information about the efficacy, cost-effectiveness, acceptability and feasibility of a novel way to improve type 2 diabetes self-management.

## Methods/design

### Study design

The study is a two-arm prospective RCT in which a total of 340 adults with type 2 diabetes will be randomised to either the intervention (TLC Diabetes program) or 'routine care' control arm. Participants in both arms complete assessments at baseline (Time 1), 6-month follow-up (Time 2), and 12-month follow-up (Time 3).

### Study aims

*Primary Aim*: To investigate the effects of the TLC Diabetes program on health outcomes (primary outcome variables include HbA1c and QoL measured using the Short Form-36 version 2 [SF-36v2]) post intervention (Time 2) and at 12- month follow-up (Time 3).

*Secondary Aim*: To examine the cost-effectiveness of the intervention arm in comparison with the control arm.

### Study sample

#### Eligibility criteria

Eligibility criteria include: a type 2 diabetes diagnosis of at least 3 months; aged 18 - 70 years; residing in the greater Brisbane area (Australia); an HbA1c level of at least 7.5%; stable diabetes pharmacotherapy type for at least 3 months; stable pharmacotherapy dosage for at least 4 weeks; ability to clearly speak and understand English via the telephone, and weekly access to a telephone. Participants are excluded if they are: diagnosed with a condition with likely poor prognosis within 1 year; diagnosed with dementia or a psychiatric co-morbidity; pregnant, lactating, or planning to become pregnant within the next 12 months; currently enrolled in another intervention trial; or have undergone bariatric surgery in the past 2 years.

#### Sample recruitment procedures

A range of recruitment strategies are used including advertisements in newspapers and community newsletters and distribution of flyers to a large number of health professionals to provide to patients diagnosed with type 2 diabetes. Patients of diabetes clinics of three major hospitals in Brisbane and clients of Diabetes Australia - Queensland's shops and information seminars are also informed of the study by research staff or by flyers placed in reception areas.

Individuals expressing interest in participating are screened in two stages. They are firstly assessed against eligibility criteria at first contact via telephone or in person. If deemed potentially eligible, they attend a baseline appointment at one of two large teaching hospitals, the Princess Alexandra Hospital (PAH) or the Royal Brisbane and Women's Hospital (RBWH) in Brisbane, where they are provided with a comprehensive explanatory statement. After signing the study consent form, they complete the baseline questionnaires and a hospital phlebotomist takes the blood specimens required. They are excluded if the HbA1c result from this blood test does not meet the HbA1c criterion.

#### Sample size calculations

A total of 340 eligible participants will be recruited to the study. It is anticipated that there will be an attrition rate of up to 30% over the 12 months of follow-up, so we expect complete data on 238 participants. With 238 completing participants, we shall be able to detect, with 90% power and type I error of 5% (two-tailed), excess intervention effects over routine care effects of at least 0.4% (from baseline 8.9% to 8.5%) in HbA1c. The calculations for HbA1c were based on standard deviations of change of 1% in both groups, conservatively estimated assuming maximum physiological range of 3% improvement or deterioration of individuals. An effect size of 0.4% in our primary outcome, HbA1c, was chosen for our sample size calculations as per Toobert [18], who calculated that a change of 0.4% translates into a clinically meaningful 14% reduction in risk of diabetes complications based on the analysis of the UK Prospective Diabetes Study in patients with type 2 diabetes. For SF-36v2 physical and mental component summary scores, a sample of 238 completed participants has 90% power to detect a difference of 3.9 units (assuming standard deviation of 10 [19]).

### Ethics approval

Ethics approval was received from Human Research Ethics Committees of the Princess Alexandra Hospital (No. **2007/029**), Royal Brisbane and Women's Hospital (No. **HREC/09/QRBW/21**), Prince Charles Hospital (No. **HREC/O9/QPC HJ26**), the University of Queensland (No. **2007000899**) and Monash University (No. **CF07/0313 - 2007/0102**).

### Study arms

All participants receive a quarterly newsletter consisting of general health information; this aims to maintain participation.

#### Control arm

Control participants are advised to continue their routine medical care.

#### Intervention arm

Intervention participants also continue their routine medical care. In addition, they receive the TLC Diabetes program which was developed collaboratively between the Australian research team and USA researchers at the Medical Information Systems Unit, Boston University [20,21]. The TLC Coordinator, whose role is to support intervention group participants in all aspects of use of the TLC Diabetes program, meets with participants within one week of their Time 1 data collection to provide them with the TLC Diabetes kit containing the TLC Handbook, an *ACCU-CHEK*^® ^*Advantage *glucose meter, test strips, and a Bluetooth™ device with which to upload their blood glucose results to the TLC Diabetes system. The TLC Handbook contains instructions and general information on the TLC Diabetes program, a number of Diabetes Australia diabetes management fact sheets plus sheets on which to record notes and information related to the program. A quit smoking information pack, containing a self-help booklet and brochures, is also provided to current smokers. During this meeting, participants also receive instructions on how to operate the glucose meter and the uploading Bluetooth™ device and complete a training call to the TLC Diabetes system. Participants are asked to perform all blood glucose self-monitoring with the study glucose meter and to upload its readings immediately preceding their weekly telephone conversations with the TLC system.

Participants choose a unique personal password which they enter at the start of each call to the system and which is linked to their database file and ensures correct subject identification and confidentiality. Prior to the participants' first call to the system the TLC Coordinator obtains personalised self-care clinical targets for the participants from their primary health care provider. This includes recommended number of daily blood glucose tests, ideal fasting blood glucose range and clearance for physical activity. Once this information is entered into the database, participants start making weekly calls to the system over a period of 24 weeks. The calls are at no cost to them and they can last between 5 and 20 minutes (depending upon the content of the call and the participant's responses). Blood glucose monitoring is the first topic covered in each weekly call. It is followed by one of the following topics: medication-taking (calls 1-4; 13-16), physical activity (calls 5-8; 17-20), and healthy eating (calls 9-12; 21-24). When a participant does not take any medication prescribed for diabetes, the medication-taking topic is replaced by physical activity. When the treating physician does not provide clearance for physical activity, this topic is replaced by medication-taking. In cases when there is no clearance for physical activity and no pharmaceutical treatment of diabetes, the participant does not hear a second topic on calls 1-8, and 13-20.

The TLC Coordinator phones participants after their first two calls to the TLC system and at weeks 6, 12 and 20, to identify and resolve any issues faced during their use of the TLC Diabetes system or to identify reasons for not calling regularly. Additionally, the TLC Diabetes system sends email "alerts" to a dedicated project email address to signal the need for the Coordinator to contact a participant regarding technical or other issues.

### Study integrity

The study design is according to the recommendations of the CONSORT statement for randomised trials of non-pharmacologic treatment [22]. Randomisation to an experimental group occurs after the Time 1 baseline assessment is completed. Arm allocation is conducted using a 4 × 4 block randomised block design with the participant as the unit of randomisation. Due to the complex nature of the intervention, it is not possible to blind research staff to group allocation. The intervention protocol is documented and the data generated during the calls made by participants is stored in the TLC database. All analyses will be conducted based on the principle of intention to treat.

### Measurement

All assessments (Table 1) are administered at the PAH or RBWH but at the same location for Time 1, 2 and 3. Blood specimens are collected according to Queensland Health guidelines. Waist and hip circumferences are measured according to WHO MONICA guidelines [23]. Behaviour, psychosocial and health care utilisation (HCU) questionnaires are self-administered.

**Table 1:** Primary and secondary outcome measures for times 1, 2 and 3

Variable	Instrument
**Primary outcome variables**	
Glycaemic control	HbA1c
Quality of life	SF-36v2 [24,25]

**Secondary outcome variables**	
Blood lipids	Total cholesterol, LDL cholesterol, HDL cholesterol, triglycerides
Insulin sensitivity	HOMA score
Kidney function	Creatinine and estimated Glomerular Filtration Rate (e-GFR)
Blood pressure	Measured twice using Welch-Allyn electronic sphygmomanometer on same arm. A third measure is taken when the first two readings differ by more than 10 mmHg and 6mmHg for systolic and diastolic blood pressure respectively.
Body Mass Index (BMI)	Calculated from height and weight measured by research staff
Waist and hip circumference	Measured by research staff according to WHO MONICA project guidelines [23]
Diet	Anti Cancer Council of Victoria Food Frequency Questionnaire [30] (ACCVFFQ)
Physical activity	Active Australia Survey [29]
Self-efficacy (nutrition and physical activity)	Self-efficacy scales [28]
Anxiety and depression	Hospital Anxiety and Depression Scale [26]
Social support	ENRICHD Social Support Inventory [27]
Smoking	Self-report
Adherence to recommendations for blood glucose testing, foot-care and medication	Summary of Diabetes Self-Care Activities [31]
Health care service utilisation, except hospital admissions	Self-report for visit to health professionals, usage of hospital services not involving admissions and medications
Hospital admissions	Electronic records maintained by Queensland Health, plus self-report for admissions to private hospitals
Co-morbidities	Self-report

#### Primary and secondary outcome variables

Primary outcome variables are: HbA1c and health-related QoL measured by the SF-36v2 [24,25]. Secondary outcome variables include: clinical measures (blood lipid profile, body mass index [BMI], waist and hip circumferences, blood pressure), psychosocial outcomes (depression and anxiety symptoms [26], social support [27]), nutrition and physical activity self-efficacy [28], physical activity [29], diet [30], adherence to foot-care, medication taking and blood glucose testing [31]. Information pertaining to HCU (visits to health practitioners, hospital admissions, other hospital services and medications), healthcare costs and costs of the intervention is also collected. The measures for these variables are summarised in Table 1.

#### Intervention implementation

Adherence to the program is assessed by the proportion of completed calls to the system relative to the expected number of calls, time interval between calls, and call duration. In addition, satisfaction with and perception of usefulness of the intervention are assessed by self-administered questionnaire at Time 2 and a semi-structured interview at Time 3.

#### Socio-demographic variables

Self-reported socio-demographic variables include gender, age, ethnicity, marital status, education, employment status, private health insurance status and household income.

### Data analyses

Assessment of similarity of baseline characteristics across randomised groups will be performed using appropriate summary statistics. If any imbalances of characteristics between the two groups are identified, these will be adjusted for in the main analytical modelling as supplementary analyses. Evaluation of the TLC Diabetes intervention effect will be based on an intention-to-treat analysis. Analysis of covariance (with baseline score as the covariate) will be fitted to estimate differences by intervention group in changes over time at each time point. Analysis across all time points using all available data, including as much as possible those of participants lost to follow-up, will employ generalised estimating equations. Results will be expressed as estimated mean changes in primary and other outcome variables by group, and as overall mean excess intervention over routine care effects, all with corresponding 95% confidence intervals.

### Cost-effectiveness analysis

Detailed economic data will be collected throughout the trial to enable a comprehensive evaluation of the intervention's efficiency when compared to routine care. Data on HCU will be obtained from participants at times 1, 2 and 3 and data on all public hospital admissions will be obtained from Queensland Health. Standard costs will be applied to HCU in both arms (e.g. Australian-Revised Diagnostic Related Groups for hospital admission costs). The intervention arm will also incur the costs of the intervention including set-up costs that will be annuitized (e.g. computer, telephone line connections) and operating costs (e.g. TLC Coordinator).

Within-trial and modelled over the rest of life cost-utility analyses will be undertaken from the perspective of direct health care costs to the government. SF-36v2 scores will be converted to utility weights using the SF-6 D algorithm [32] for the calculation of quality-adjusted life years (QALYs) - the primary outcome for the economic evaluation. The incremental costs and QALYs will be calculated as the differences between participants in the intervention and routine care groups. The resulting incremental cost-utility ratio will provide a measure of the relative value for money of the intervention using the additional cost per QALY gained. One-way and probabilistic sensitivity analyses will be undertaken for all parameters with uncertainty and/or variability [33].

## Discussion

Previous trials of automated TLC systems targeting physical activity, nutrition and medication adherence have demonstrated the effectiveness of such technology to improve health behaviours and chronic disease self-management [13,16,17]. To date, this innovative and accessible form of self-management support program has not been formally tested in relation to diabetes management and its cost-effectiveness has not been examined. Therefore, this study will provide valuable information on the effectiveness, user-acceptability and feasibility of this telehealth system. It addresses the need to investigate new approaches to deliver ongoing and regular diabetes self-management support to relieve health systems from the growing demands caused by the increasing prevalence of diabetes around the world.

## Competing interests

The authors declare that they have no competing interests.

## Authors' contributions

BO and RHF conceived the original study design and its development. BO, RHF, AR, SA, MDC, RW, IS, PS and DB developed the intervention and study protocols. DB and MC drafted the manuscript. All authors read and contributed to the final manuscript.

## Pre-publication history

The pre-publication history for this paper can be accessed here:

http://www.biomedcentral.com/1471-2458/10/599/prepub

## References

[B1] Shaw JE Sicree RA Zimmet PZ Global estimates of the prevalence of diabetes for 2010 and 2030 Diabetes Research and Clinical Practice 2010 87 1 4 14 10.1016/j.diabres.2009.10.007 19896746

[B2] International Diabetes Federation IDF Diabetes Atlas 2009 4 Brussels, Belgium: International Diabetes Federation 35914061

[B3] UK Prospective Diabetes Study (UKPDS) Group Intensive blood-glucose control with sulphonylureas or insulin compared with conventional treatment and risk of complications in patients with type 2 diabetes (UKPDS 33) The Lancet 1998 352 9131 837 853 10.1016/S0140-6736(98)07019-6 9742976

[B4] Selvin E Marinopoulos S Berkenblit G Rami T Brancati FL Powe NR Golden SH Meta-Analysis: Glycosylated Hemoglobin and Cardiovascular Disease in Diabetes Mellitus Annals of Internal Medicine 2004 141 6 421 431 15381515 10.7326/0003-4819-141-6-200409210-00007

[B5] American Diabetes Association Standards of Medical Care in Diabetes-2010 Diabetes Care 2010 33 Supplement 1 S11 61 20042772 10.2337/dc10-S011 PMC2797382

[B6] Norris SL Engelgau MM Narayan KMV Effectiveness of self-management training in type 2 diabetes: a systematic review of randomized controlled trials Diabetes Care 2001 24 3 561 587 10.2337/diacare.24.3.561 11289485

[B7] Norris SL Lau J Smith SJ Schmid CH Engelgau MM Self-management education for adults with type 2 diabetes: a meta-analysis of the effect on glycemic control Diabetes Care 2002 25 7 1159 1171 10.2337/diacare.25.7.1159 12087014

[B8] Fisher EB Brownson CA O'Toole ML Anwuri VV Ongoing follow-up and support for chronic disease self-mangement in the Robert Wood Johnson Foundation Diabetes Initiative The Diabetes Educator 2007 33 Supplement 6 201S 207S 10.1177/0145721707304189 17620402

[B9] Murray E Burns J See Tai S Lai R Nazareth I Interactive Health Communication Applications for people with chronic disease Cochrane Database of Systematic Reviews 2005 4 10.1002/14651858.CD004274.pub4 PMC13184810 16235356

[B10] Balas EA Krishna S Kretschmer RA Cheek TR Lobach DF Boren SA Computerized knowledge management in diabetes care Medical care 2004 42 6 610 621 10.1097/01.mlr.0000128008.12117.f8 15167329

[B11] Jackson CL Bolen S Brancati FL Batts-Turner ML Gary TL A systematic review of interactive computer-assisted technology in diabetes care. Interactive information technology in diabetes care Journal of General Internal Medicine 2006 21 2 105 110 16390512 10.1111/j.1525-1497.2005.00310.x PMC1484664

[B12] Krishna S Balas EA Boren SA Maglaveras N Patient acceptance of educational voice messages: a review of controlled clinical studies Methods of Information in Medicine 2002 41 5 360 369 12501806

[B13] Delichatsios HK Friedman RH Glanz K Tennstedt S Smigelski C Pinto BM Kelley H Gillman MW Randomized trial of a "talking computer" to improve adults' eating habits American Journal Of Health Promotion 2001 15 4 215 224 11349340 10.4278/0890-1171-15.4.215

[B14] Farzanfar R Frishkopf S Migneault J Friedman R Telephone-linked care for physical activity: A qualitative evaluation of the use patterns of an information technology program for patients Journal of Biomedical Informatics 2005 38 3 220 228 10.1016/j.jbi.2004.11.011 15896695

[B15] Glanz K Shigaki D Farzanfar R Pinto B Kaplan B Friedman RH Participant reactions to a computerized telephone system for nutrition and exercise counseling Patient Education and Counseling 2003 49 2 157 163 10.1016/S0738-3991(02)00076-9 12566210

[B16] Jarvis KL Friedman RH Heeren T Cullinane PM Older women and physical activity: using the telephone to walk Women's Health Issues 1997 7 1 24 29 10.1016/S1049-3867(96)00050-3 9009864

[B17] Pinto BM Friedman R Marcus BH Kelley H Tennstedt S Gillman MW Effects of a computer-based, telephone-counseling system on physical activity American Journal of Preventive Medicine 2002 23 2 113 120 10.1016/S0749-3797(02)00441-5 12121799

[B18] Toobert DJ Glasgow RE Strycker LA Barrera M Radcliffe JL Wander RC Bagdade JD Biologic and quality-of-life outcomes from the Mediterranean Lifestyle Program: a randomized clinical trial Diabetes Care 2003 26 8 2288 2293 10.2337/diacare.26.8.2288 12882850

[B19] Jenkinson C Stewart-Brown S Petersen S Paice C Assessment of the SF-36 version 2 in the United Kingdom Journal of Epidemiology and Community Health 1999 53 1 46 50 10.1136/jech.53.1.46 10326053 PMC1756775

[B20] Bird D Oldenburg B Wootton R Friedman R Development and evaluation of an automated telephone system for diabetes self-management [abstract] Annals of Behavioral Medicine 2008 35 Supplement S013

[B21] Oldenburg B Bird D Friedman RH Adaptation of an automated telephone system to promote physical activity in Australia [abstract] Annals of Behavioral Medicine 2005 29 Supplement S083

[B22] Boutron I Moher D Altman DG Schulz KF Ravaud P Extending the CONSORT Statement to randomized trials of nonpharmacologic treatment: explanation and elaboration Annals of Internal Medicine 2008 148 4 295 309 18283207 10.7326/0003-4819-148-4-200802190-00008

[B23] WHO MONICA Project. MONICA manual, part III: population survey, section 1: population survey data component, 1997 http://www.ktl.fi/publications/monica/index.html

[B24] Ware JE Kosinski M Dewey J How to score version 2 of the SF-36 Health Survey 2000 Lincoln, RI: QualityMetric Incorporated

[B25] Ware JE Sherbourne CD The MOS 36-item short-form health survey (SF-36): I. Conceptual framework and item selection Medical Care 1992 30 6 473 483 10.1097/00005650-199206000-00002 1593914

[B26] Zigmond AS Snaith RP The Hospital Anxiety and Depression Scale Acta Psychiatry Scandinavia 1983 67 6 361 370 10.1111/j.1600-0447.1983.tb09716.x 6880820

[B27] Mitchell PH Powell L Blumenthal J Norten J Ironson G Pitula CR Froelicher ES Czajkowski S Youngblood M Huber M A short social support measure for patients recovering from myocardial infarction: the ENRICHD Social Support Inventory Journal Of Cardiopulmonary Rehabilitation 2003 23 6 398 403 10.1097/00008483-200311000-00001 14646785

[B28] Schwarzer R Renner B Social-cognitive predictors of health behavior: action self-efficacy and coping self-efficacy Health Psychology 2000 19 5 487 495 10.1037/0278-6133.19.5.487 11007157

[B29] Australian Institute of Health & Welfare The Active Australia Survey: A guide and manual for implementation, analysis and reporting Canberra 2003

[B30] Hodge A Patterson AJ Brown WJ Ireland P Giles G The Anti Cancer Council of Victoria FFQ: relative validity of nutrient intakes compared with weighed food records in young to middle-aged women in a study of iron supplementation Australian and New Zealand Journal of Public Health 2000 24 6 576 583 10.1111/j.1467-842X.2000.tb00520.x 11215004

[B31] Toobert DJ Hampson SH Glasgow RE The Summary of Diabetes Self-Care Activities measure: Results from 7 studies and a revised scale Diabetes Care 2000 23 7 943 950 10.2337/diacare.23.7.943 10895844

[B32] Brazier J Roberts J Deveril M The estimation of a preference-based measure of health from the SF-36 Journal of Health Economics 2002 21 2 271 292 10.1016/S0167-6296(01)00130-8 11939242

[B33] Doubilet P Begg CB Weinstein MC Braun P McNeil BJ Probablistic sensitivity analysis using Monte Carlo simulations: a practical approach Medical Decision Making 1985 5 2 157 177 10.1177/0272989X8500500205 3831638

